# Case report of severe constrictive perimyocarditis and ischemic hepatitis in a Crohn’s disease patient upon infliximab-induced lupus-like syndrome

**DOI:** 10.1177/17562848211044033

**Published:** 2021-09-29

**Authors:** Simon Hirschmann, Sarah Fischer, Entcho Klenske, Katharina Dechant, Jörg H.W. Distler, Christoph Treutlein, Markus F. Neurath, Raja Atreya

**Affiliations:** Department of Medicine 1, Friedrich-Alexander-University Erlangen-Nürnberg, University Hospital Erlangen, Germany; Deutsches Zentrum Immuntherapie (DZI), Friedrich-Alexander-University Erlangen-Nürnberg, Erlangen, Germany; Department of Medicine 1, Friedrich-Alexander-University Erlangen-Nürnberg, University Hospital Erlangen, Germany; Deutsches Zentrum Immuntherapie (DZI), Friedrich-Alexander-University Erlangen-Nürnberg, Erlangen, Germany; Department of Medicine 1, Friedrich-Alexander-University Erlangen-Nürnberg, University Hospital Erlangen, Erlangen, Germany; Deutsches Zentrum Immuntherapie (DZI), Friedrich-Alexander-University Erlangen-Nürnberg, Erlangen, Germany; Department of Medicine 2, Friedrich-Alexander-University Erlangen-Nürnberg, Erlangen, Germany; Department of Medicine 3, Friedrich-Alexander-University Erlangen-Nürnberg, Erlangen, Germany; Deutsches Zentrum Immuntherapie (DZI), Friedrich-Alexander-University Erlangen-Nürnberg, Erlangen, Germany; Department of Radiology, Friedrich-Alexander-University Erlangen-Nürnberg, Erlangen, Germany; Department of Medicine 1, Friedrich-Alexander-University Erlangen-Nürnberg, University Hospital Erlangen, Erlangen, Germany; Deutsches Zentrum Immuntherapie (DZI), Friedrich-Alexander-University Erlangen-Nürnberg, Erlangen, Germany; Department of Medicine 1, Friedrich-Alexander-University Erlangen-Nürnberg, University Hospital Erlangen, Ulmenweg 18, 91054 Erlangen, Germany; Deutsches Zentrum Immuntherapie (DZI), Friedrich-Alexander-University Erlangen-Nürnberg, Erlangen, Germany

**Keywords:** anti-TNF antibody, case report, Crohn’s disease, infliximab, lupus-like syndrome

## Abstract

Anti-tumor necrosis factor (TNF) antibodies have become an indispensable part in the therapeutic landscape of treating inflammatory bowel disease (IBD) patients. Nevertheless, they can be associated with the occurrence of severe systemic side effects. Here, we report the case of a 23-year-old patient with ileocolonic Crohn’s disease in endoscopic remission under ongoing anti-TNF infliximab therapy with occurrence of novel generalized arthralgia, pleuritic chest pain, and dyspnea. Clinical, laboratory, and imaging diagnostic workup in an extended clinical routine setting at the University Hospital of Erlangen, Germany, was used by a multidisciplinary team consisting of gastroenterologists, radiologists, cardiologists, and rheumatologists to investigate the underlying cause of the clinical symptoms in the patient. The results received using the aforementioned diagnostic setup led to the diagnosis of severe constrictive perimyocarditis due to infliximab-induced lupus-like syndrome with distinct ANA reactivity and elevated anti-dsDNA levels. Furthermore, pronounced ischemic hepatitis was diagnosed. Infliximab treatment was immediately stopped, and initiated corticosteroid pulse therapy only led to partial response as it had to be reduced due to pronounced psychiatric side effects. Persistent signs of pericarditis required additional ibuprofen therapy, which led to subsequent resolution of cardial symptoms. Formerly elevated liver enzymes returned to normal, and there were no clinical signs of recurrence of Crohn’s disease activity over 18 months of follow-up. The patient was subsequently switched to ustekinumab therapy for further treatment of underlying Crohn’s disease. This case report describes for the first time severe infliximab-induced lupus-like syndrome in an IBD patient, concurrently mimicking ST-elevation myocardial infarction with MRI visualization of pericarditis, occurrence of ischemic hepatitis, and pronounced signs of systemic inflammation.

## Introduction

The substance class of tumor necrosis factor (TNF) inhibitors represents an established and effective option in the therapeutic algorithm of treating inflammatory bowel disease (IBD) patients. Although there is a frequent induction of autoantibodies upon anti-TNF therapy,^1^ the development of autoimmune diseases such as drug-induced lupus (DIL) is a rather rare event.^2,3^ The clinical and laboratory features of an anti-TNF-induced lupus-like syndrome (ATIL) resemble idiopathic systemic lupus erythematosus (SLE). The diagnosis of DIL is made upon development of antinuclear antibodies (ANA) and double-stranded DNA (dsDNA) antibodies in conjunction with the most common clinical symptoms, such as arthralgia, serositis, fever, or myalgia. A predominant portion of patients also presents skin involvement, such as malar rash.^4^ Renal, central nervous, or gastrointestinal complications are rare.^4–6^ Here, we present the case of a Crohn’s disease patient who presented himself with severe constrictive perimyocarditis due to infliximab-induced lupus-like syndrome. CARE guidelines have been followed for this case report. A signed informed consent form for publication was obtained by the patient.

## Case

A 23-year-old male patient with a 7-year history of ileocolonic Crohn’s disease presented himself to our emergency department with progressive generalized arthralgia, pleuritic chest pain, dyspnea, and tachycardia, which initially had started 3 weeks ago. There has been no anamnestic history of a viral prodrome or infection, nor was there any initial laboratory or clinical sign of a viral infection. An initial physical examination showed no specific findings, particularly no pericardial or pleural rub. The patient’s underlying Crohn’s disease was in clinical and endoscopic remission under therapy with the anti-TNF antibody infliximab, which had been started 11 months ago. There had never been any extra-intestinal joint manifestations in the patient’s course of disease. The patient presented no morning stiffness and no signs of joint swelling with an exacerbation of the arthralgia following physical stress and toward the evening without any correlation of pain with a specific position.

The initial laboratory results presented elevated C-reactive protein levels of 248.4 mg/l (normal: <5 mg/l) without an increase in procalcitonine. Correlating with these findings, the white blood cell count was increased (15.2 × 10^3^/µl, 79.7% neutrophils). The initial platelet count was normal (283 × 10^3^/µl), and hemoglobin was below the lower limit of normal (12.7 g/dl). Moreover, creatinine, aspartate aminotransferase (AST), bilirubin, coagulation parameters, and lipase were within the normal range, and Gamma-glutamyl transferase (GGT) was slightly elevated with 85 U/l.

Further rheumatological laboratory findings demonstrated distinct ANA reactivity (1:3200) and elevated anti-dsDNA levels (18 U/ml) without C3 or C4 complement consumption. Anti-histone antibodies presented negative. The patient showed no kind of cytopenia. There were no signs of mucocutaneous or renal involvement. IgM, IgG, and IgG4 were within the normal range.

To rule out further differential diagnoses, virological testing was performed, showing no abnormalities; in particular, an cytomegalovirus polymerase chain reaction (PCR) test in the blood was negative.

The diagnosis of ATIL due to treatment with the anti-TNF antibody infliximab was made by interdisciplinary evaluation with the rheumatological department according to the American College of Rheumatology (ACR)/European League Against Rheumatism (EULAR) criteria at the time. The previous therapy with infliximab was immediately decided to be stopped.

Two days after admission from the emergency department, the patient presented himself with acute cold sweat, tachycardia, and mild hypotension.

**Figure 1.**
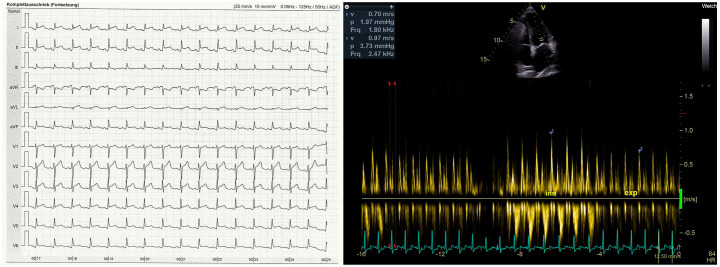
Left: Initial 12-channel ECG of our patient with ST elevations in I, II, aVF, andV2–V6 as well as ST depression in aVR. Right: Transthoracic echocardiography showing the transmitral inflow profile (pulsed wave Doppler) with significant respiratory variance as a sign of hemodynamic relevance of pericardial effusion.

A 12-lead electrocardiogram (ECG) demonstrated ST elevations in I, II, aVF, and V2–V6 as well as ST depression in aVR (Figure 1 left). Transthoracic echocardiography demonstrated reduced systolic global function with a significantly reduced longitudinal shortening (lateral and anterior) and a circular pericardial effusion (10 mm end diastolic), resulting in a hemodynamic compromise (Figure 1 right), matching the aforementioned clinical presentation. A contrast-enhanced computed tomographic (CT) scan of the thorax and abdominal region was also performed, where small pericardial and pleural effusions could be detected.

**Figure 2.**
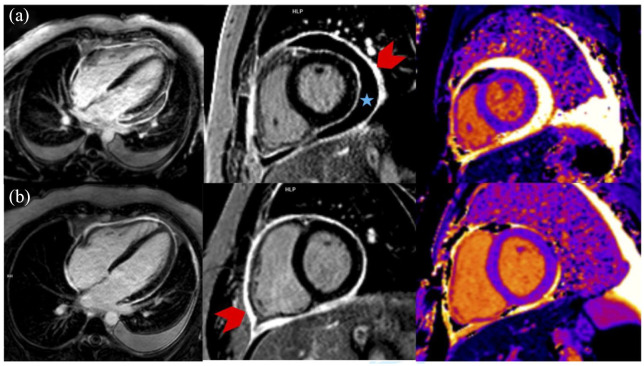
Cardiac MRI: late gadolinium enhancement (LGE) in four-chamber view and short-axis view (SAX). T1 relaxation times as color-coded maps (T1maps) in SAX. Pericardial effusion (blue asterisk) and pericardial LGE (red arrow) indicating pericarditis (a; 2018/08). Increased pericardial thickening and LGE with decrease in pericardial effusion (b; 2019/01). Absent myocardial LGE and normal relaxation times in T1maps, so there are no more signs of previous myocardial involvement. Note the left-sided pleural effusion.

The patient was transferred to the intensive care unit, and a coronary angiography performed due to suspected ST-elevation myocardial infarction ruled out the presence of coronary stenosis. Constrictive pericarditis upon ATIL was treated with systemic steroid therapy (initial 60 mg prednisolone/day). Within 24 h after onset of cardial symptoms, the patient also presented serological signs of a pronounced non-cholestatic hepatitis [AST 5478 U/l, alanine aminotransferase (ALT) 4011 U/l, glutamate dehydrogenase (GLDH) 1796 U/l, GGT 132 U/l, bilirubin 1.3 mg/dl], which in combination with marked elevation of lactate dehydrogenase (LDH) levels (5391 U/l) were interpreted as an ischemic-type hepatitis following preceded hypoperfusion and hemodynamic compromise. An acute viral hepatitis was ruled out by PCR testing (types A, B, C) and serology (type E) in the blood. A heart magnetic resonance imaging (MRI) scan showed no signs of myocarditis. Transesophageal echocardiography ruled out the presence of possible endocarditis. The patient immediately reported relief of his thoracic pain and arthralgia upon initiation of glucocorticoid treatment. However, the daily prednisolone dose had to be reduced to 30 mg/day, as the patient developed glucocorticoid-induced psychiatric side effects. One week after starting the glucocorticoid therapy, AST and ALT values were nearly normalized and CRP levels were markedly diminished. A follow-up MRI scan of the heart 1 week later, which was done due to recurrent thoracic pain and tachycardia, described progressive pericarditis with new onset of inflammation in the myocardial tissue (Figure 2, row a).

In addition to ongoing glucocorticoid therapy (prednisolone 30 mg/day), concomitant ibuprofen treatment (1.8 g/day) was initiated. The patient quickly responded to this therapy, showing significant improvement in thoracic pain. He was discharged at his personal request against medical advice. At this time, liver-associated laboratory values had normalized. Five months later, another follow-up cardiac MRI was performed. It still showed an increased pericardial thickening with persistent signs of pericarditis. However, MRI morphological signs of improvement could be detected, represented by a decrease in pericardial effusion and resolved myocardial affection (Figure 2, row b). Ustekinumab therapy for treatment of the patient’s Crohn’ disease, which had remained in remission, was subsequently initiated 5 months after discharge since a reinitiation of infliximab was contraindicated due to the life-threatening course of associated systemic lupus–like syndrome. An earlier start of the recommended ustekinumab therapy was declined by the patient, who was justifying this with his clinical remission. All cardiological follow-up examinations over a period of 18 months, including transthoracic echocardiography, showed normal results.

## Discussion

Unlike in SLE, there are currently no specific diagnostic criteria existing for DIL.^6–8^ This impedes early and definite diagnosis, which is indispensable for the initiation of specific therapies. According to the existing literature, the diagnosis of ATIL should be considered in patients receiving anti-TNF therapy and, in a temporal relationship, developing at least one non-serological (including arthritis, serositis, hematological disorder, or malar rash) and one serological (such as ANA or anti-dsDNA positivity) criterion for lupus according to the ACR.^6,7^

There is no difference in sex distribution in DIL. Up to 95% of all DIL patients suffer from arthralgia/arthritis, whereas only 2% show malar/acute cutaneous rash and less than 25% present with Raynaud’s phenomenon. Also, in contrast to SLE, renal, central nervous system, and hematological involvement in DIL represents a scarcity. Regarding serological findings, low complement levels are rare; moreover, less than 45% of the DIL patients show anemia and only 2–33% leukopenia.^4^

In our patient, the development of arthralgia and polyserositis with associated clinical symptoms, as well as the de novo appearance of elevated ANA and anti-dsDNA levels, 11 months after initiating an anti-TNF infliximab therapy, supported early diagnosis of ATIL. The onset of symptoms in this case is also compatible with current studies, describing predominant clinical manifestation of ATIL in a time range of 10 days to 54 months after commencing anti-TNF therapy.^9^ Regarding ATIL, the disappearance of associated symptoms is described in 94% of the cases within 3 weeks to 6 months after cessation of anti-TNF therapy.^6^ Autoantibody production, however, may persist for several months after the discontinuation of the causative drug agent.^5^ The therapeutic need for corticosteroids and eventually additional immunosuppressive therapy, such as methotrexate, azathioprine, leflunomide, cyclophosphamide, or mycophenolate,^6^ should give rise to the suspicion of unmasking an underlying pre-existent SLE.

In particular for IBD, it is known that both Crohn’s disease and ulcerative colitis share common genetic variants with SLE.^10^ Moreover, IBD has shown to be associated with SLE in large-scale cohorts.^11^ In this case, our patient also fulfilled the current criteria for the diagnosis of SLE according to the ACR and EULAR. ANA development following anti-TNF therapy is common.^12^ According to prospective rheumatoid arthritis cohorts, 31–63% of patients treated with infliximab and 16–51% of patients treated with adalimumab developed ANA.^4^ In contrast, only few patients develop ATIL. Postmarketing studies describe the estimated prevalence of DIL, with 0.19–0.22% for infliximab, 0.18% for etanercept, and 0.10% for adalimumab.^13^ According to an international registry of autoimmune disorders induced by biologics (BIOGEAS registry), DIL occurred in 0.33% of patients after exposure to biologics, with a higher frequency in patients treated with infliximab.^14^ Disproportionality analyses (case/non-case method) performed based on a French pharmacovigilance database calculated higher reporting odds ratios (RORs) for infliximab (10.97) and adalimumab (9.03) than for etanercept (ROR 4.02) regarding the association between specific anti-TNFα antibodies and the occurrence of DIL.^15^

The underlying pathogenic mechanisms explaining the development of DIL are not completely understood.^4^ In this context, the predominant portion of available studies is related to hydralazine and procainamide. Proposed mechanisms linked to the pathogenesis of DIL include genetic predisposition, drug biotransformation, and epigenetic dysregulation on T and B cells, neutrophils, and macrophages, possibly causing autoreactive T- and B-cell generations linked to DIL,^3,16^ although it needs to be emphasized that the underlying pathomechanism differs between the substance classes. A significant amount of data investigates the inhibition of DNA methyltransferases and thus of epigenetic silencing by procainamide and hydralazine.^17^

However, recent data increasingly describe strong associations between DIL and newer agents, such as TNF inhibitors.^4^ By inhibiting Th1 response, anti-TNFα agents could be assumed to trigger Th2 autoimmune reactivity, including SLE.^5^ Pathogenic hypotheses for the ATIL syndrome include a reduced CD44 expression influencing the clearance of apoptotic neutrophils and nuclear debris by phagocytes, thereby leading to autoantibody production against nuclear antigens and DNA. As a second pathogenic mechanism, the activation of polyclonal B lymphocytes stimulating autoantibody production is proposed, following increased infection rates due to immunosuppression by anti-TNFα agents. A third hypothesis states that anti-TNFα agents cause suppression of Th1 immune responses, generating a ‘cytokine shift’ in the direction of Th2 cytokine production, like interleukin (IL)-10 and interferon (IFN)-α, promoting humoral autoimmunity.^6^ Further studies are warranted to better characterize the underlying pathogenic mechanisms. Since most available data regarding ATIL contain small cohorts and often report on patients with mild disease and few clinical manifestations,^6^ the management of severe cases of ATIL remains challenging and requires interdisciplinary efforts to optimize patient outcomes. To our knowledge, only four other cases of severe constrictive pericarditis or cardiac tamponade in association with infliximab therapy in IBD patients have been described.^8,18–20^ Two^8,18^ of these four case reports have diagnosed ATIL as the underlying pathomechanism. However, to our knowledge, our case report describes for the first time the appearance of ATIL in an IBD patient, mimicking ST-elevation myocardial infarction with MRI visualization of pericarditis, occurrence of ischemic hepatitis, and pronounced signs of inflammation.

The risk of recurrence of ATIL has been reported to be 69% when using the same anti-TNFα agent again and 29% when another anti-TNF therapy is started.^7^ We therefore stopped infliximab therapy in the patient and initiated therapy with the IL-12/IL-23 inhibitor ustekinumab, which successfully maintained remission of the patient’s Crohn’s disease.
